# Data Protection by Design in the Context of Smart Cities: A Consent and Access Control Proposal

**DOI:** 10.3390/s21217154

**Published:** 2021-10-28

**Authors:** Said Daoudagh, Eda Marchetti, Vincenzo Savarino, Jorge Bernal Bernabe, Jesús García-Rodríguez, Rafael Torres Moreno, Juan Antonio Martinez, Antonio F. Skarmeta

**Affiliations:** 1 CNR-ISTI, 56124 Pisa, Italy; eda.marchetti@isti.cnr.it; 2 Engineering Ingegneria Informatica S.p.A., 90146 Palermo, Italy; vincenzo.savarino@eng.it; 3 Department of Information and Communication Engineering, University of Murcia, 30100 Murcia, Spain; jorgebernal@um.es (J.B.B.); jesus.garcia15@um.es (J.G.-R.); rtorres@um.es (R.T.M.); juanantonio@um.es (J.A.M.); skarmeta@um.es (A.F.S.)

**Keywords:** access control, consent manager, GDPR, privacy-by-design, smart cities

## Abstract

The growing availability of mobile devices has lead to an arising development of smart cities services that share a huge amount of (personal) information and data. Without accurate and verified management, they could become severe back-doors for security and privacy. In this paper, we propose a smart city infrastructure able to integrate a distributed privacy-preserving identity management solution based on attribute-based credentials (p-ABC), a user-centric Consent Manager, and a GDPR-based Access Control mechanism so as to guarantee the enforcement of the GDPR’s provisions. Thus, the infrastructure supports the definition of specific purpose, collection of data, regulation of access to personal data, and users’ consents, while ensuring selective and minimal disclosure of personal information as well as user’s unlinkability across service and identity providers. The proposal has been implemented, integrated, and evaluated in a fully-fledged environment consisting of MiMurcia, the Smart City project for the city of Murcia, CaPe, an industrial consent management system, and GENERAL_D, an academic GDPR-based access control system, showing the feasibility.

## 1. Introduction

Nowadays, the wide availability of mobile devices and applications has increased the adoption and diffusion of Smart Information and Communication Technology (ICT) Systems (SISs), such as smart homes, smart cities, and smart campuses. On the one hand, SiSs represent an effective improvement for users’ daily life and work. On the other hand, they cause the generation, analysis, management, and sharing of enormous quantities of information. Indeed, data about the movement of people, the places visited, or services requested are continuously circulating between SIS services and users. This is raising not only problems about the volume, range, and granularity of the data collected and processed, but also about privacy and trustworthiness risks.

Even if the latest applicable European Regulations (e.g., the General Data Protection Regulation (GDPR) [1]) are currently forcing SISs to be more accountable regarding security and privacy, there are still some issues regarding which data should be managed under the umbrella of such European Regulations. Some examples are information coming from Global Positioning System (GPS), sensors, and cameras. These devices collect and share personal data (in some cases sensitive), which can expose SISs to security attacks by malicious hackers and privacy vulnerability breaches.

Considering in particular Smart Cities (SCs), guaranteeing the *Privacy of Location* [2] becomes a fundamental aspect to avoid that an external actor could capture sensitive information about users’ identity and behaviors. The situation could be even more critical in the case cameras or radio communication systems (e.g., BLE and Wi-Fi, or magnetic sensors) are used in combinations with GPS for a more accurate users localization. In this context, the commonly adopted technical solutions for privacy preservation could not fully avoid the indirect identification of different users and their behaviors.

For better figuring out the problem, without losing generality, let us consider a user moving inside an SC providing features such as navigation services for optimizing paths, looking for parking or shopping areas, queuing management service, notification of user’s utilities (for instance electricity or water consumption), and advertising services for special offers or sales. Even if extremely simple and appealing, these few features hide at least three significant drawbacks:

Controller point of view. In collecting the GDPR consents, the controller should explicitly consider a more generic concept of personal data. Data such as MAC or IP addresses, subject’s localization with relative date and time, storage of the visited locations, devices used, personal preferences…Should be considered personal data and therefore processed in compliance with the GDPR’s provisions. Additionally, due to the appealing amount of data sharing, it could be possible that competitors would unlawfully exploit the SCs environment for collecting information for increasing their commercial solutions, selling their specific products or facilities, or the different users’ behaviors. Controllers should avoid such situations and ensure that subjects’ information is correctly authorized, managed, stored, and protected by all the entities involved in the SC environment.
Data subject point of view. The appealing facilities that an SC makes available can encourage the subject to accept the GDPR consents without a clear understanding of the usage and exploitation of the collected data. Thus, the necessity to define a user-centric, privacy-by-design SC arises. The different purposes of the processing of personal data must be explicitly stated through specific and non-ambiguous consent. Additionally, they should also indicate the characteristics of the collection and protection of personal, and how they enforce the rights of the data subject.
Authority point of view. In SC, the Authority shall ensure the accountability principle, i.e., demonstrating compliance with the other GDPR principles. When more than one (external) service is included in the SC environment, assuring this principle may become challenging. Thus, SC should enforce specific governance procedures for demonstrating that all the components and services of the SC environment can ensure the required level of data protection.

As from this simple example, hidden in the golden world of SC, full of interesting, useful, and appealing features, there is an enormous amount of (personal) data that subjects and services are leaving in the different (third parties) databases without being completely aware of the risk.

In this situation, even if SCs are putting in practice successful data management for most of the security threats and vulnerabilities, there are still some issues about the data privacy protection. Enforcing authorized and secure data access control does not assure *per se* the lawful treatment of the different kinds of (personal) data that are circulating inside the SCs. Consequently, the privacy risks may turn into a reduction of individual trust in the global SC environment.

In this paper, we propose an extended SC architecture to enforce and assure *by-design* different GDPR provisions such as (i) the definition of specific purpose, collection of data, and specification of consents for processing personal data; (ii) the enforcement of access control to personal data; and (iii) the data subject exercising her/his rights. For this, we focused on an enhanced privacy-preserving system, based on p-ABCs, with integrated id proving and mobile eID (from eIDAS) that potentiates usability and trust across the SC services. In this way, the proposed enhanced architecture ensures that citizens and companies can manage and track personal data in a straightforward, user-centric, and user-friendly manner.

The solution relies on security services, i.e., authorization systems (i.e., Access Control (AC)), enhanced with specific features to enforce additional legal requirements, such as the data usage purpose, user consent, and the data retention period. Additionally, the joint work of the consent and security services assures the controller’s and processor’s compliance with the regulation and the generation of machine-readable policies directly from the General Data Protection Regulation (GDPR)’s rules, avoiding error-prone management.

  The remainder of the paper is organized as follows. Section 2 presents the basic concepts used along the proposal and related works; Section 3 describes the proposed solution; Section 4 shows the proof-of-concept we implemented by instantiating the proposed solution with real artifacts coming from both industrial and academic contexts; whereas in Section 5 we illustrate the reference architectural flows; the performance of the proposal is discussed in Section 6; and finally Section 7 concludes the paper, discusses the obtained results and illustrates future works.

## 2. Background and Related Work

Among the various SISs, the paper focuses on the Smart Cities (SCs) ones. To this purpose, we firstly describe the main concepts related to Smart Cities, the GDPR, Consent Management, and AC and their related works; then, we briefly present our proposal, which will be discussed in the remainder of the paper.

### 2.1. Smart Cities

In a smart city domain, several services are offered by the platform, usually corresponding to specific areas of the City Council. Those services deal with heterogeneous information, from citizens’ personal information to measurements provided by Internet of Things (IoT) devices for traffic management, or irrigation status of gardens, to name a few. The interconnection of these services implies an increase in the attack surface and a more significant impact on privacy, which can ultimately affect citizens’ safety. In addition, interoperability, lightweight protocols, and cryptographic algorithms must also be considered throughout smart cities life-cycles. To cope with these features, holistic approaches are needed to address the technical, social, and legal requirements of a smart city. In that direction, projects such as SynchroniCity  (https://synchronicity-iot.eu, accessed on 25 October 2021) have developed and addressed architectures that have been conceived within the scope of other EU research projects.

The exchange of information integrated into a smart city platform requires the development of tools that empower citizens to manage their security and privacy [3]. Nevertheless, because of the high number of devices usually integrated into this sort of platform, automated mechanisms are required to fulfill this process. A lack of this kind of tools can prevent users from specifying their consent and providers from defining the purpose of their data processing. For this reason, traditional access control technologies must be evolved so that access policies can define how the information is generated by a data provider and how it is going to be used by data consumers.

Finally, there is another aspect to be highlighted. Using standardized mechanisms for the aforementioned tools is essential so that the user’s preferences on her/his data can be easily integrated in smart city platforms. What is more, these mechanisms should be flexible to react upon configuration changes, considering the legal principles of current data protection regulations, such as the GDPR, and their evolving nature.

### 2.2. General Data Protection Regulation

The General Data Protection Regulation (GDPR) [1] is the European Union Law (Regulation) for the protection of personal data. The GDPR defines personal data as any information relating to an identified or an identifiable natural person called data subject. That means that a data subject is a Natural Person (a living human being) whose data are managed by a Controller.

The regulation is applied to the processing of personal data, whether it is automated (even partially) or not, and defines the following demands and principles:

- Transparency—i.e., data must be processed fairly, lawfully and transparently;
- Purposes—i.e., data should only be collected for determined, explicit and legitimate purposes, and should not be processed later for other purposes;
- Minimization—i.e., the data processed must be relevant, adequate and limited to what is necessary in view of the purposes for which they are processed;
- Accuracy—i.e., the data processed must be accurate and up-to-date regularly;
- Retention—i.e., the data must be deleted after a limited period;
- Subject explicit consent—i.e., the data may be collected and processed only if the data subject gives his explicit consent.

Hence, introducing the GDPR in the context of SC means not only to prevent vulnerabilities that several cyberattacks may cause but also to deal with the data privacy of people moving in smart cities and using its services.

Recently, different proposals have been conceived for making the SC architecture secure and privacy-preserving. They can be roughly divided into the following categories:

- Proposals providing supporting facilities for transforming the GDPR’s text into executable access control policies. In this case, the policies are either systematically derived from the GDPR, e.g., [4,5] or generated through intermediate formal structures [6,7].
- Proposals easily enforceable into the Smart ICT Systems architectures [8,9]. They can be divided into: (i) those using access control mechanisms for the protection of personal data within Smart ICT Systems perimeters [10]; (ii) those using Smart ICT Systems users location information for authenticating the customer and managing his/her data [11]; and (iii) those exploiting specific security attributes for assuring the GDPR compliance [12,13].

All the above proposals share the identical drawback; even if effective, they do not fully fulfill the needs of a privacy-by-design and user-centric architecture for smart cities. Differently, we propose to provide a privacy-friendly SC architecture, having the following peculiarities:

- it enables the definition of specific purposes of personal data processing;
- it provides a user-centric interface for collecting Data Subjects consents;
- it lawfully manages the Spatio-temporal information about a person’s social life;
- it allows a secure and privacy-preserving access to personal data; and finally,
- it facilitates data subjects in exercising their rights and modifying or withdrawing the already given consents.

### 2.3. Consent Manager

One of the aims of the GDPR is to empower individuals and give them control over their personal data, and consent is the legal basis to support that control. According to the GDPR (https://gdpr-info.eu/art-4-gdpr/, accessed on 3 September 2021), consent is a “freely given, specific, informed and unambiguous indication of the data subject’s wishes by which he or she, by a statement or by a clear affirmative action, signifies agreement to the processing of personal data relating to him or her”. In general, consent authorizes data sources to provide data to the data consumer and authorizes the data requester to process that data. Consent has to refer to a data usage policy that can be linked to a consent formalization. Consent needs to be given in a clear manner so that the data controller can demonstrate that valid consent has been given. In particular consent record should demonstrate: (a) who consented (b) when they consented (c) what was consented (d) how consent was given (c) whether a consent withdrawal occurred.

A consent manager has the goal to support the entire life-cycle of consent management. It enables the data subjects to trace and manage the given consents in a transparent manner. It also allows the controllers to use consents to data sharing among digital services using personal data and meet the GDPR requirements. To do that, a consent manager needs to address specific requirements of user-centeredness, transparency, standardization, and interoperability. In this work, among the different proposals focused on providing a structured, simplified and machine-readable formalization of a data processing consent, we refer to the Kantara Initiative specification (https://kantarainitiative.org/download/7902/, accessed on 26 July 2021). In this case, a consent receipt is a notice created from a record of consent provided to an individual the moment a person agrees to the collection, use, and sharing of personal information. Interoperability is guaranteed by the use of shared vocabularies such as the Data Privacy Vocabulary (DPV) (https://dpvcg.github.io/dpv/, accessed on 26 July 2021), and ontology (relationships) serialized using semantic-web standards.

One of the reference architecture is the one proposed by [14] that integrates data security and semantic descriptions into a trust-query framework, enabling the provision of user consent as a service by using the MyData approach (https://mydata.org/wp-content/uploads/sites/5/2020/08/mydata-white-paper-english-2020.pdf, accessed on 6 September 2021).

### 2.4. Access Control Concepts

Access Control (AC) is usually implemented through an *Access Control Mechanism (ACM)*, which is the system that provides a decision to an authorization request, typically based on predefined *Access Control Policy (ACP)*. eXtensible Access Control Markup Language (XACML) [15] is one of the most widely used AC languages. It provides a reference architecture in the AC environment including components like the Policy Administration Point (PAP), which is in charge of managing the policies, or the Policy Decision Point (PDP), which evaluates the policy against the request and returns the authorization decision. An XACML policy is a specific statement of what is or is not allowed. It is based on a set of rules that define conditions on attributes of subjects, resources, actions, and environment.

To interact with XACML-based AC mechanisms, subjects need means to prove their attributes so the policies can be evaluated. This is directly linked to the issue of digital identity management. Traditional technologies such as X.509 certificates or Single-Sign-On systems (e.g., based in OAuth [16]) are lacking in privacy. The former violates the minimal disclosure principle (as everything within the certificate must be revealed), whereas the latter leads to identity providers that can track users and control their data. Privacy-Enhancing Attribute-Based Credentials (p-ABC) systems mitigate these issues. They follow the same approach as X.509 certificate but allow presentations that disclose only the minimum necessary information. What is more, the user can decide whether different presentations will be linkable by revealing (or not) identifying data.

Although p-ABC systems are not new, the level of implementation and impact of even the most well-known solutions such as Identity Mixer [17] or U-Prove [18] is quite low. EU-H2020 projects such us Attribute-based Credentials for Trust (ABC4Trust) (https://abc4trust.eu/, accessed on 26 October 2021) or ReliAble euRopean Identity EcoSystem (ARIES) (https://www.aries-project.eu/, accessed on 26 October 2021) have aimed to address existing problems and improve the performance of credential systems but their adoption has been limited.

In this paper, we leverage our previous work while also taking into account the GDPR’s demands to improve our access control mechanisms. In particular, we rely on ObLivious identitY Management for Private and User-friendly Services (OLYMPUS) [19], which aims to provide a user-friendly and distributed identity management system that addresses the data minimization challenge in online and offline scenarios while maintaining good usability, implementation, and scalability features. The distributed IdP introduced in OLYMPUS allows users to leverage efficient p-ABCs.

Summarizing, our contribution with respect to the current research environment can be identified as:

- An integrated usage of the access control mechanism for enforcing the GDPR provisions, which relies on the vision presented in [20]. Therefore, the access control mechanism becomes a means for restricting access to personal data based on Access Control Policies (ACPs) compliant with the GDPR. More precisely, the considered ACPs contain a set of rules that specify who (e.g., Controller, Processor or Data Subject) has access to which resources (e.g., Personal Data) and under which circumstances (e.g., the GDPR’s demands).
- An integration of a specific Consent Management System allowing data subjects to exercise their rights and Controllers to manage the purpose and consent transparently.
- An implementation of identity proving mechanisms through mobile eID using the eIDAS infrastructure, then integrated with privacy-preserving Attribute-Based Credentials. The zero-knowledge approach enables secure access management while applying the precepts of minimal disclosure and being GDPR compliant.

## 3. Reference Architecture for Smart-Cities

**Figure 1:**
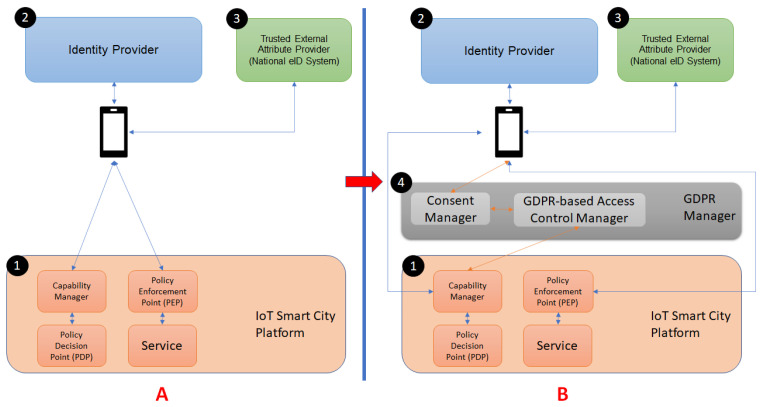
Privacy-By-Design Concept.

In this section, we present a Privacy-by-Design Generic Smart System Architecture that enables data protection by-design in the context of Smart-cities (SCs). For this, in Figure 1, we depict, on the left-hand side, the standard simplified architecture of the SC, and on the right-hand side our enhanced view.

As in Figure 1A, Smart Cities are composed of a Core System (component ① in the figure, named IoT Smart City Platform) that offers the main functionalities to Smart Services in terms of both hardware and smart software (e.g., Cloud Computing, Internet of Things, and Big Data). Developers use these functionalities to conceive and implement Smart Services, represented in the figure by the rectangle named Service, that end-users can use to achieve a given business or personal needs.

The management of the resource and data access is usually delegated to an Access Control system. As detailed in Section 2.4, it mainly includes three different components: Capability Manager, Policy decision point (PDP), and Policy Enforcement point (PEP). Thus, the Capability Manager generates capability tokens that bestow authorization to use specific services, relying on the PDP for decision making. Then, the PEP controls access to the services, checking that the request includes a valid capability token (i.e., the request is authorized). Two additional components are usually needed for user interaction with this system: The Identity Provider (component ② in Figure 1) and the Trusted External Attribute Provider (component ③ in Figure 1). These components allow potential users to prove that they fulfill the required access control policy. In this case, the Identity Provider certifies attributes for users by either generating the required attributes or aggregating attributes coming from other Identity Providers (i.e., Trusted External Attribute Providers).

Considering the original architecture, the proposal of this paper is to leverage extra components that form an additional layer for actions related to GDPR such as the user consent, as reported in Figure 1B, component ④. The GDPR Manager is in charge of modeling and enforcing the GDPR legal framework and includes two specific components: *Consent Manager* that translates the textual consent into structured representation, and *Access Control Manager* that provides enforceable access control policies. More details about the GDPR Manager components are provided in the remainder of this section.

### 3.1. Consent Manager

The Consent Manager aims to manage and control personal data during the interaction among Data Subjects and public and private services that act as Data Controllers and Processors (e.g., PA, Social, IoT, B2C). It provides facilities for lawful data sharing processes, with the ability to grant and withdraw consent to third parties for accessing personal data. Concerning Smart Services, the Consent Manager allows them to define specific purposes for each operation (i.e., processing activities) and the data needed to accomplish the required tasks lawfully.

Consequently, the Consent Manager should include a consent-based, user-centric interface enabling:

(1) data subjects to manage and trace their data and its associated consent;
(2) controllers to specify specific purposes for processing personal data; and
(3) controllers/processors to use the given consent for data sharing among digital services using personal data and meet the GDPR’s requirements.

Additionally, the Consent Manager should guarantee by-design compliance with the GDPR’s demands, such as data minimization and purpose limitation principles.

### 3.2. Access Control Manager

The Access Control Manager has the responsibility of creating ACPs that are compliant by-design with the GDPR. It works in collaboration with the Consent Manager by receiving, as input, the machine-readable specification of services definitions and the related Data Subjects’ consents. More precisely, the Access Control Manager component uses Personal Data related to Data Subject classified in categories as required by the GDPR; information about the Controller of each service and the defined purposes; the consent given by the Data Subject in terms of a relation between Personal Data and Purposes. Based on that information, the Access Control Manager can create specific Access Control Policies (ACPs), each related to an article of the GDPR. The peculiarities of the Access Control Manager are the possibility to (a) be integrated with different Consent Managers and (b) collaborate with different Access Control systems.

## 4. Instantiation of the Architecture in the Mimurcia Smart City Context

In this section, we describe a possible implementation of the enhanced Smart City architecture proposed in Figure 1B by considering the following components, coming from both industrial and academic contexts: the MiMurcia IoT Platform for the Smart City system (see Section 4.1); CaPe for the Consent Manager (see Section 4.2); and customization of the GENERAL_D framework for the GDPR-based access control policies derivation (see Section 4.3).

### 4.1. Mimurcia Iot Platform

**Figure 2:**
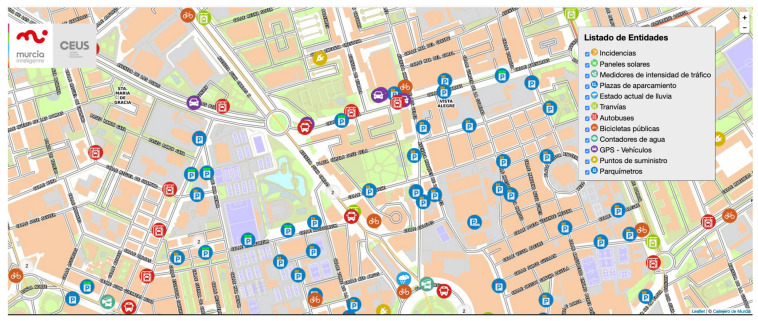
MiMurcia viewer.

MiMurcia is the name of the Smart City project for the city of Murcia, in the southeast of Spain. This ambitious project has the goal of bringing the city of Murcia to the Smart City environment. To do so, it has considered the integration of heterogeneous services such as public transport (tram, bus, and bicycles), solar panel energy information, traffic information, parking meters, parking sites, street lighting, smart irrigation of parks and gardens, noise sensors, weather stations among others. In a first iteration of this project, MiMurcia has already integrated some of these services as presented in the map-based viewer of Figure 2.

For this purpose, MiMurcia is based on a FIWARE-based IoT platform, exploiting all the advantages of both NGSI API and data model to represent heterogeneous information and expose this information using an open and standard API. This platform comprises several modules, each of them taking an important and specific task. From the IoT level, there is an IoT backend responsible for translating and tailoring the information represented using lightweight protocols such as CoAP, MQTT, or Ultralight into the NGSI data model. All this information arrives into the core component of this platform, an NGSI broker exposing both query/response and publication/subscription approaches for distributing and sharing the information with other modules. Since this broker is only responsible for having the current view of the information, i.e., it is not aimed at providing historical information about the entities, this broker is usually linked to another module in charge of providing this historical series of data. Other components are also connected with the broker to provide open and transparent information to the citizens by using an Open Data Portal. Big data exploitation is also considered in this platform, where, thanks to another component called Cygnus, this information can be transmitted to a Hadoop file system to be exploited with big data techniques. Other services, such as a Geographic Information System (GIS) are connected so that web viewers can be geo-located in a map-based website, allowing the presentation of different layers of information which can be of interest for the city council staff or the citizens.

Last, security has been integrated as a cornerstone aspect to control access to the information stored in this platform. A key element for this purpose is the identity manager, responsible for managing the information of the entities that can take part in the process of obtaining or introducing data to the platform. This component is also responsible for the authentication process. This technology is complemented with a distributed and fine-grained access control solution based on the XACML framework and policy definition. It enables the definition of flexible access control policies in terms of the subject which is issuing the request, the resource he intends to access, and the operations he would like to perform (i.e., reading or writing operations).

The MiMurcia infrastructure has been further extended with the inclusion of the OLYMPUS (More information about the system, like details on its architecture, can be found in [19]) identity management system (IdM) for privacy-preserving authentication of users. OLYMPUS IdM has multiple advantages over traditional identity providers (IdP). The IdP role is distributed, reducing the risk of impersonation and forgery attacks. Also, it employs privacy-preserving Attribute-Based Credentials (p-ABCs). P-ABCs can be used for generating proofs over user attributes, revealing only information necessary for the specific transaction (minimal disclosure). What is more, multiple uses of a credential are unlinkable unless the user decides to reveal identifying data. Thus, p-ABCs are a fitting tool for privacy-preserving attribute-based control. Another advantage is that the IdP can no longer track user activities, like which services she uses or when. Lastly, this specific deployment relies on eIDAS as an attribute source. This adds trust in the solution, as there are strong ties to the user’s physical identity.

**Figure 3:**
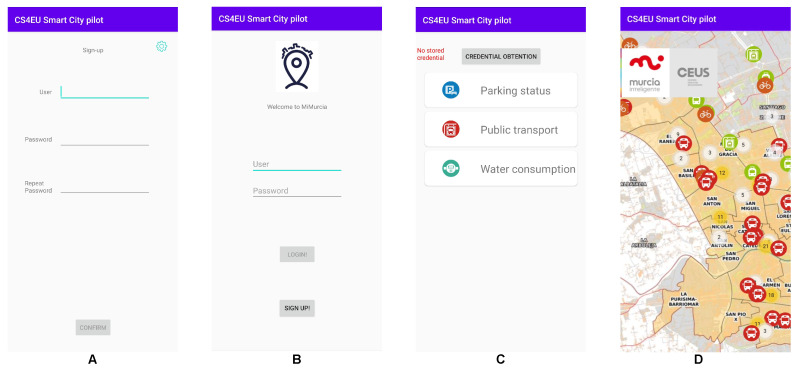
MiMurcia IoT Platform: proof of concept application screens.

A mobile application serves as a proof of concept of the user interaction with the MiMurcia platform, relying on the OLYMPUS vIdP for privacy-preserving authorization. This application offers three information services with different access requirements: parking status, public transport information, and data on the water consumption of public facilities. The application has screens dedicated to three main processes, **enrolment**, **credential issuance**, and **accessing platform services**. The Sign-Up screen (Figure 3A) allows users to choose their username and password for the vIdP and perform enrolment. The Login screen (Figure 3B) enables the retrieval of new credentials. The Home screen (Figure 3C) enables service selection and dictates the access process, up to the point where the service is actually being used (Figure 3D).

**Figure 4:**
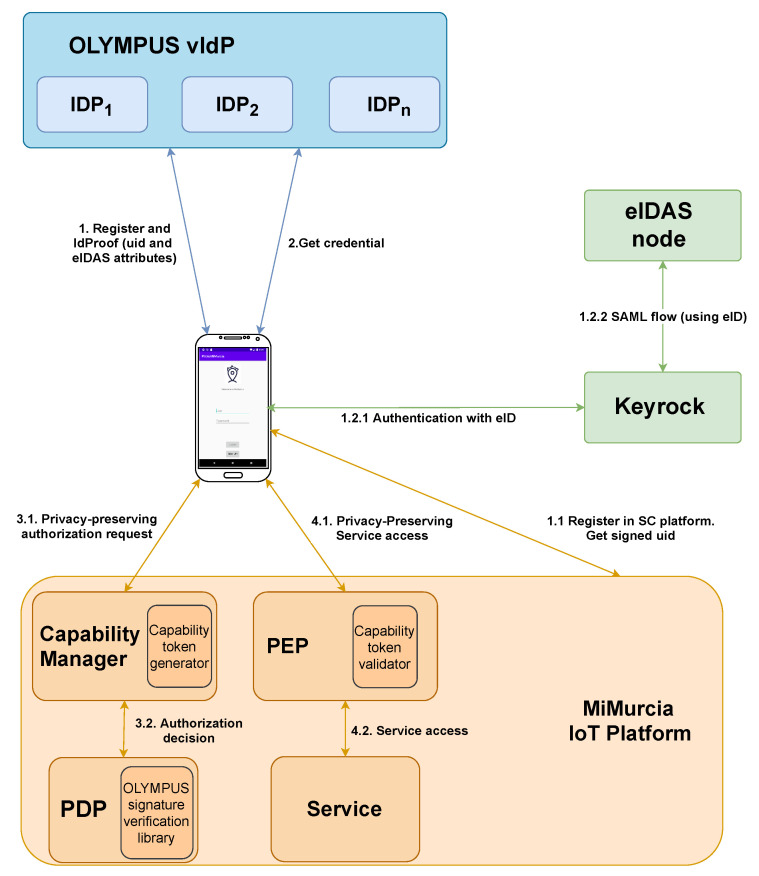
MiMurcia IoT Platform: Components and Flow.

Figure 4 depicts the flows that occur during the mentioned interactions and how the components interact, centered on the point of view of the user’s application. During enrolment (steps 1.X), the SC platform will create a new user profile (i.e., register the user in the platform) and return an assertion of the associated user id. A new user account in the vIdP will also be created. That account will be populated with the attributes certified by the eIDAS framework (the user must authenticate against a node using an eID, with Keyrock being used as a bridge that helps with the interaction), as well as the platform user id. Note that this will only happen if the corresponding assertions are successfully verified by the vIdP.

After they have enrolled, users can obtain a credential (step 2) by authenticating against the vIdP. A valid authentication will give the user a freshly issued p-ABC that holds all her attributes and maybe (securely) stored. Note that, as the user id for the SC platform is one of the attributes contained in the credential, the user will be able to authenticate against the SC by generating a zero-knowledge proof that reveals said id. As an added feature, she will also have the option of remaining anonymous in other transactions by keeping the id hidden (because of the unlinkability of p-ABC presentations).

Lastly, the user can access platform services. The process can be divided into two sub-processes, authorization (steps 3.X) and actual service access (steps 4.X). During the former, the user has to prove that she meets some requirements to be authorized (and receive the corresponding capability token) to use the service using p-ABC zero-knowledge presentations. Before proceeding to the proof, the user must consent to revealing the requested data to the platform. In the latter, the PEP checks that the request is authorized (that is, a valid capability token is submitted) and only then allows service usage.

### 4.2. Cape

CaPe is the selected Consent Manager implementation that we adopted in the instantiation of the proposed architecture. CaPe provides an open-source ICT suite for consent-based, user-centric personal data management during the interaction among data subjects and public and private services in order to collect privacy preferences as informed and explicit consents. CaPe follows the MyData principles to exploit the potential of personal data, facilitates its control and new business opportunities in compliance with the GDPR.

**Figure 5:**
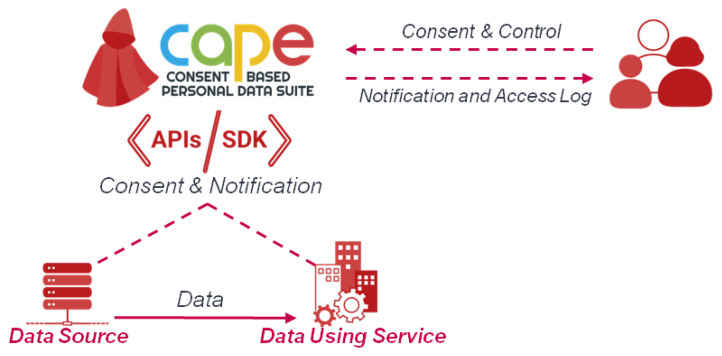
Overview of CaPe.

**Figure 6:**
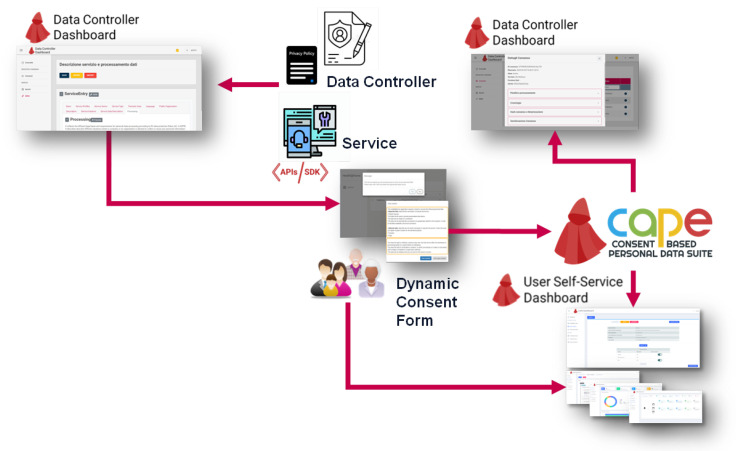
How CaPe Works.

In this frame, CaPe acts as an intermediary and as a tool of communication between data subjects and controllers/processors, supporting the generation and management of dynamic consents (Figure 5). A general CaPe use is provided in Figure 6. In the depicted scenario, through the *Data Controller Dashboard* an organization can model the legal basis for the processing of personal data: in a standardized manner; in accordance with the relevant information (i.e., purpose, processing, and type of data); and in line with the related privacy policy.

According to the derived model, CaPe automatically generates the consent form that can be shown to the data subject in order collect the selected preferences. The CaPe consent manager module stores the data subject preferences in a standard, open and machine-readable format. The two separated dashboards can let, on one side, the Data Controller to view and manage all the consents collected, on the other, the Data Subject, through the *User Self-Service Dashboard*, to check which data is used, how and for what purpose and to manage the related consents.Any modification of consents is traced, notified and the new privacy preferences are forwarded to the services involved in personal data processing.

### 4.3. GENERAL_D

GENERAL_D (Gdpr-based ENforcEment of peRsonAL Data) is an integrated solution for defining and developing AC systems that guarantee by-design the compliance with the GDPR [20]. GENERAL_D consists of:

- A GDPR-based Life Cycle for authorization systems, which defines a specific and integrated process development life cycle for the specification, deployment, and testing of adequate fine-grained authorization mechanisms taking into account legal requirements.
- An integrated environment for automatically enforcing the data protection or privacy regulation for specifying the privacy requirements, controlling personal data, processing them, and demonstrating compliance with the GDPR in collecting, using, storing, disclosing, or disposing of the personal data.

The GENERAL_D abstract architecture can be customized with several tools and methodologies for assisting the development of GDPR-based Access Control Systems by following the principle of Data Protection by design and Data Protection by default.

In leveraging AC systems as a technical means for protecting “personal data by-design” and gaining legal compliance with the GDPR, GENERAL_D contributed to the following challenges:

1. Defining a GDPR-based Life Cycle for authorization systems and a reference architecture, enabling data protection by-design aiming at the following peculiarities:
  - Generalization—an abstract specification of the different life cycle’s activities has been proposed. Activities have been abstracted, i.e., for each of them, only what the activity expects at the beginning in terms of input and the obtained result (i.e., the expected output) has been defined.
  - Flexibility—to provide a solution that gives the GENERAL_D users (e.g., an SME) the possibility to implement the different phases most suitably and profitably.
  - Adaptability—to provide an adaptable solution to the different GENERAL_D users’ needs in terms of both available methodologies and technologies.
  - Cost Reduction—to provide the GENERAL_D users a (semi-)automated solution for their compliance needs and the possibility to include/reuse (already) existing technologies.
2. Defining a GDPR profile for a standardised AC language;
3. Defining a systematic approach for gathering and developing ACPs compliant-by-design with the regulation;
4. Advancing the notion of Data Protection Backlogs by introducing specific User Stories focused on the GDPR provisions and their technical requirements;
5. Enabling an Agile development of ACSs;
6. Defining a comprehensive testing framework for validating both GDPR-based and traditional ACSs;
7. Promoting the application of ACSs in different contexts.

**Figure 7:**
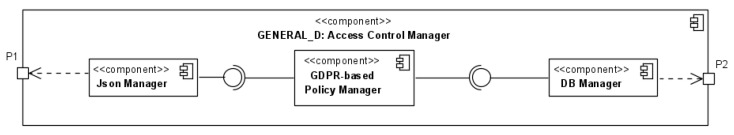
Overview of GENERAL_D Access Control Manager.

Here, we have provide specific customization of the GENERAL_D framework for obtaining GDPR-based access control policies directly from the consent provided by CaPe to allow lawful processing of personal data managed by the MiMurcia IoT Platform. The customization of GENERAL_D includes three main components (see Figure 7): Json Manager; GDPR-based Policy Manager; and DBs Manager.

**Figure 8:**
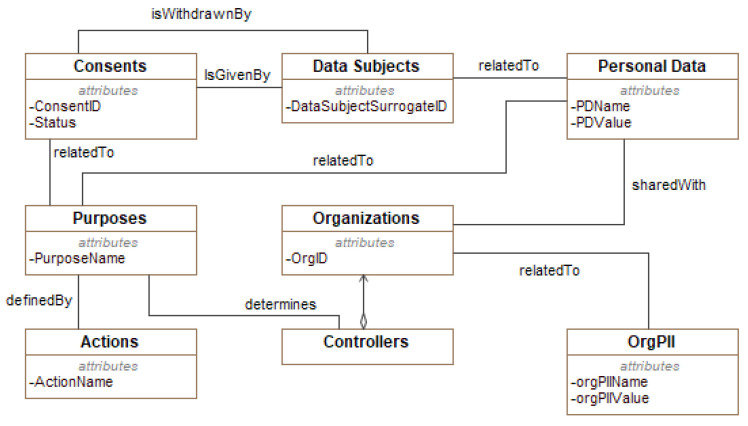
CaPe’s Consent Model: An Extract (Adopted from [21]).

**Json Manager** has the responsibility to interact directly with CaPe described in the previous section. It receives the consent in JSON format, and it parses that consent to extract the relevant information for the ACPs generation purpose. Such information includes, among others, the *Consent ID* and the *Consent Usage Rules* that contain the defined purposes of the processing, the allowed operations, and Personal Data provided by the Data Subject.
  For confidentiality reasons, we report in Figure 8 just an extract of CaPe’s consent model. CaPe models the Consent as an entity having an identifier ConsentID) and a status (Active, Non-Active).
  A *Data Subject*, identified by her/his ID, can either give her/his consent and withdraw it at any time, as stated in Art. 7 of the GDPR (see the *withdrawnBy* association between Data Subjects and Consent entities in the figure). She/He is related to a set of *Personal Data*, each represented by a name/value pair. The *Consent* is given a specific processing *Purpose* defined by the *Controller*, each having a name and it is implemented by means a set of *Actions*.
  The Data Subject can share her/his Personal Data with one or more *Organizations*, so as the controller can eventually achieve the defined purposes.
**GDPR-based Policy Manager** is the core component of the GENERAL_D framework. It has the responsibility of creating enforceable ACPs encoded in the XACML language. It interacts with: (1) Json Manager for retrieving the data to be processed (e.g., the Controller’s data, the defined purposes, the list of allowed third parties); (2) DBs Manager to retrieve the GDPR-based ACPs templates and store the obtained policies.
**DBs Manager** offers database supporting functionalities to the ACP Manager (e.g., create/modify/delete a database, and insert/modify/delete specific entries in the available tables).

By considering an instance of the consent conforms with the CaPe-s model depicted in Figure 8, from a procedural point, as shown in Algorithm 1, through the Json Manager component, GENERAL_D parses the JSON file for retrieving the data of interest, i.e., Personal Data, Purposes, and the third parties. Then, through the collaboration of the GDPR-based Policy Manager and DBs Manager, an XACML-based policy compliant with the GDPR is generated.

In detail, considering the Algorithm 1:

- **line 1–4.** The algorithm takes as input the consent represented in JSON format (ConsJF) and parses that file by obtaining its internal representation (ConsJFAsPOJO, line 4).
- **line 5–9.** In the case of active consent (Algorithm 1, line 6), the algorithm verifies whether the processed consent is a modification of an already given one. In such a case, the related ACP derived so far is modified to DENY-ALL policy (Algorithm 1, line 8).
- **line 10–13.** For each specific purpose, an XACML access control rule is generated (Algorithm 1, line 12).
- **line 14–15.** In case of withdrawing the content, the received JSON input contains the status *non-Active* (Algorithm 1, line 14), and in terms of AC, this means that no one can access Personal Data related Data Subject. That is reflected in denying all the incoming access requests by triggering the default DENY-ALL policy, modified in Algorithm 1, line 15.

**Algorithm 1** GDPR-based ACP Derivation1:**input:**ConsJF2:**output:**GACP3:GACP←{}4:ConsJFAsPOJO←JasonManager.parse(ConsJF)5:cID←ConsJFAsPOJO.getCID()6:**if**ConsJFAsPOJO.isActive()**then**7:  **if** PolicyManager.isAlreadyGiven(cID) **then**8:    PolicyManager.DenyAllPolicy(cID)9:  **end if**10:  **Foreach** $p_{j}\in ConsJFAsPOJO$ **do**11:    $ACR\leftarrow PolicyManager.CreateACPS(p_{i},cID)$12:    GACP.add(ACR)13:  **end for**14:**else if** !ConsJFAsPOJO.isActive() **then**15:  PolicyManager.DenyAllPolicy(cID)16:**end if**17:**return**GACP▹ Consent as Json File▹ GDPR-based ACP expressed in XACML       ▹ For each Purpose▹ Create a specific rule▹ Add the rule to the current policy    ▹ Return the GDPR-based ACP related with the consent

## 5. Architectural Flows

As referred on the improved architecture presented in Figure 1B, the GDPR Manager is responsible for interacting directly with MiMurcia’s End-Users for what concerns the management of the consent to be given to both the MiMurcia IoT Platform and the offered Services.

In the current implementation, the GDPR Manager has been realized by two components: (1) CaPe, i.e., the Consent Manager, that interacts with the end-users of the system, and (2) the customization of GENERAL_D described in the previous section, that translates the CaPe’s consents into enforceable policy used by the PDP of the Murcia IoT platform.

For the aim of simplicity, from a behavioral point of view, the user interaction with the MiMurcia platform can be summarized into three main use-cases scenarios:

For the aim of simplicity, from a behavioral point of view, in the following, we summarize the user interaction with the MiMurcia platform into three main use-cases scenarios:

1. MiMurcia User is registered for the first time into the platform, from which point she/he can retrieve a credential and perform the login.
2. MiMurcia User already has credentials, and she/he chooses to use a new service.
3. MiMurcia User already has credentials, and she/he is modifying the already given consent to one of the provided services.

In the following, we describe the combination of the first and second identified scenarios since they cover all the main activities considered. For the aim of simplicity, we depict the scenario’s description as an activity diagram showing the interaction between the components of Figure 1.

**Figure 9:**
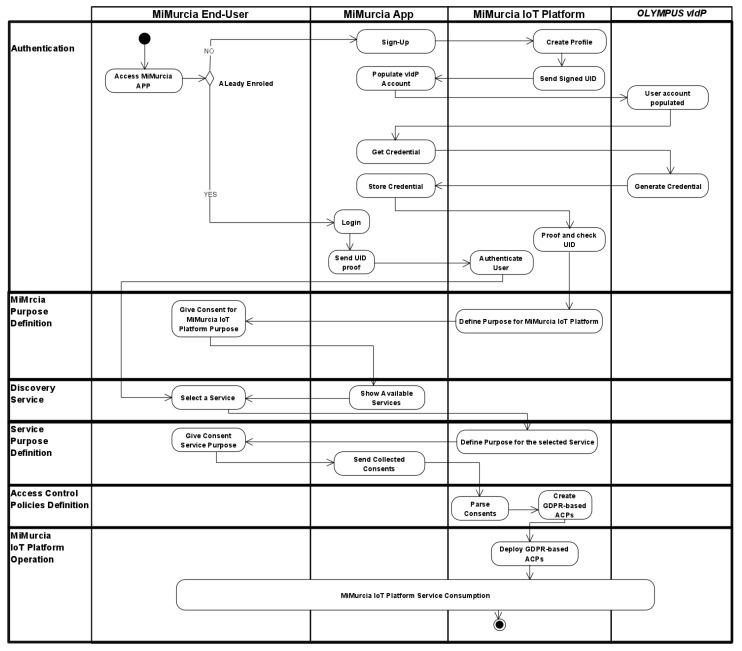
Sign-Up and Login to MiMurcia IoT Platform.

As reported in the Figure 9, six different stages have been identified:

**(1)**  **Authentication.** This concerns the interaction with users during the enrolment and login (through p-ABCs) steps as described in Section 4.1. In this stage, the registration of end-users and smart services to the MiMurcia IoT Platform is performed. In particular, MiMurcia will manage the authentication procedure of both the end-users and smart services.
**(2)**  **MiMrcia Purpose Definition.** In this stage, through the interaction with CaPe Consent Manager, the user can give the consent of the MiMurcia platform (Give Consent for MiMurcia IoT Platform Purpose activity of Figure 9 for a specific purpose (Define Purpose for MiMurcua Platform).
**(3)**  **Discovery Service.** This stage focuses on the end-users interaction with CaPe to provide specific consent and personal data for each of the different services MiMurcia IoT Platform makes available. That is the activity Show Available Services, in which the list of facilities of MiMurcia IoT Platform is shown to the user. She/He, therefore, can select one or more services through the Select a Service activity.
**(4)**  **Service Purpose Definition.** In this phase, the user gives her/his consent (Give Consent Service Purpose activity) to the selected service for processing specific personal data for a specific purpose defined by the service provider (Define Purpose for the selected Service). At this point, CaPe generates a processable consent in JSON format and sends it GENERAL_D for deriving the suitable access control policy (Send Collected Consents activity).
**(5)**  **Access Control Policies Definition.** This phase aims at translating the given consent into enforceable access control policies (Create GDPR-based ACPs activity). In this stage, during the Parse Consent activity, the GENERAL_D takes as input the JSON consents, and it generates a set of ACPs that are compliant with GDPR. Indeed, these policies are customized by using the data contained in that JSON file, e.g., purpose, consent, and personal data.
**(6)**  **MiMurcia IoT Platform Operation.** After deploying the GDPR-based access control policy, the MiMurcia End-User can start consuming the services offered by MiMurcia platform. Also, the enforcement of the GDPR’s demands encoded in the deployed ACPs is performed during the operation phase.

## 6. Performance Evaluations

In this section, we provide the performance evaluation of the different components of the proposed Proof-of-Concept architecture. In the evaluation, the integration with the CaPe application has been performed “as a service” to collect and retrieve the users’ consents. The load-balanced deployment of the CaPe consent manager allowing the interaction with the end-users does not impacted the performance of the remaining Components in the experiment run.

### 6.1. MiMurcia

The MiMurcia platform has been built using different FIWARE enablers, as well as a specific access control mechanism based on DCapBAC (capability-based access control mechanism). Previous works on the literature have evaluated these individual components with promising results (in terms of performance and scalability). For instance, components were evaluated in IoTCrawler [22], and others like [23] or [24] evaluated the whole FIWARE platform. Additionally, the performance of the DCapBAC technology has also been reviewed in another work [25].

In this paper, we evaluate the performance of MiMurcia’s infrastructure and the developed a mobile application, focusing on the processes related to access control. The measurements were carried out using a Poco X3 NFC device (Octa-core 2.3 GHz and 6 GB RAM), and each final time value was obtained as the average of ten results. It is also worthy of note that users’ identity credentials contained eleven attributes in these experiments, as the number of attributes has an impact on the cost of the involved cryptographic operations. Lastly, these measurements do not take into account user input (i.e., introducing username/password, accepting policies, …), but only the computational procedures.

**Figure 10:**
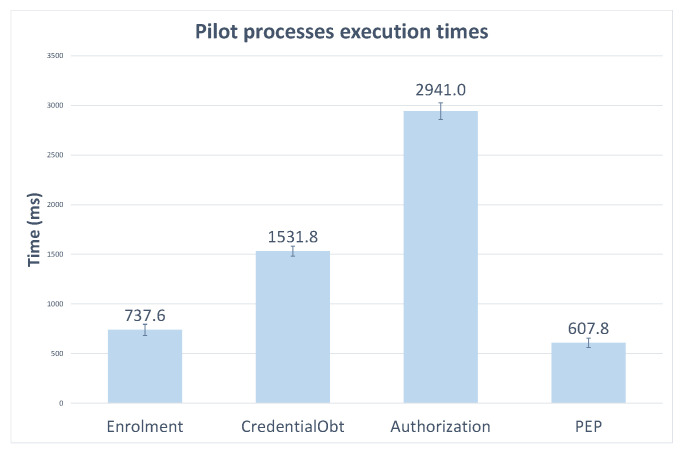
PoC implementation execution time for the different phases.

Figure 10 shows the results for the three main processes described in Section 4.1: enrolment, credential issuance, and service access. Recall that enrolment only entails creating a profile and populating the OLYMPUS account with the user’s attributes, while credential issuance involves a user login and cryptographic operations (generating and combining credential shares). However, note that the service access is broken down into two steps: getting authorization (obtaining a capability token) and accessing the service through the PEP (i.e., PEP verifying the capability token allows the operation and enabling service usage). Apart from being two differentiated operations, this separation is relevant because capability tokens have a determined lifetime in which users will be able to reuse them to access a service through the PEP directly.

In the graph, it is apparent that the authorization process is by far the heaviest. This is a natural result, as it is a complex operation, and it involves the most bulky cryptographic procedures. In any case, the execution times are not prohibitive (even less than it appears as the authorization step is only needed for some service usages and credential issuance all the more rarely) and allow a comfortable user experience.

**Figure 11:**
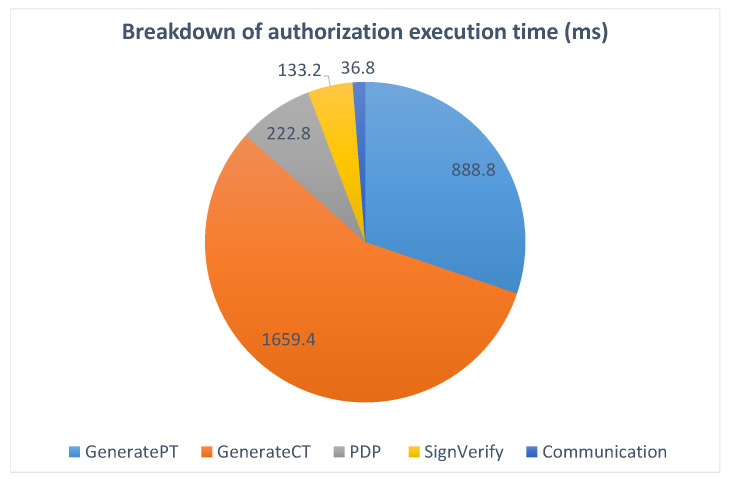
PoC implementation breakdown of authorization execution time.

Figure 11 gives a detailed breakdown of the operations executed during the authorization step, showing the proportion of time taken by each of them. The process starts when the user accepts the access policy and a presentation token conforming to it is generated (*GeneratePT*). This is one of the most costly operations as the cryptographic method behind it is also one of the heaviest within the p-ABC scheme, and it is being executed on the (more constrained) mobile phone. Next, the token is sent to the capability manager, which controls the authorization flow in the smart platform. There, the PDP is contacted for a decision on the access request (*PDP*), the validity of the token is verified (*SignVerify*), and the capability token is generated (*GenerateCT*) and sent back to the user application.

### 6.2. GENERAL_D

We have evaluated the performance of the customized version of the GENERAL_D framework in the context of Smart-cities. Therefore, we focused on the process of converting the consent provided by CaPe into an executable GDPR-based access control policy expressed in the XACML formalism.

The measurements were carried out using a Dell Laptop (Intel(R) Core(TM) i9-9980HK CPU @ 2.40 GHz and 16 GB RAM), and each final time value was obtained as the average of 20 executions. For the evaluation, different consent structures have been considered either coming from real case studies or obtained by operating specific modifications or extensions to the existing one. They differentiate each other in the complexity and in the number of, for instance, personal data and purposes considered to cover the most realistic scenarios. Consequently, by referring to the CaPe’s Consent Model depicted in Figure 8, the relevant elements affecting the structural complexity of an instance of the consent, and consequently the most changed ones, are purposes, Actions, Personal Data, and Organizations.

**Figure 12:**
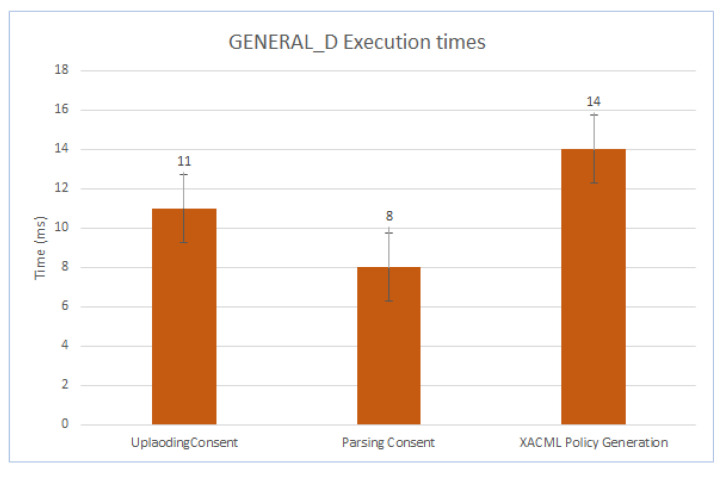
GENERAL_D Execution Time for Different Phases.

Figure 12 reports the results associated with the main three phases of the process: uploading consent, parsing the consent, and XACML policy generation. As in the figure, the overall process took 33 ms on average. Almost half of the total time refers to the XACML Policy Generation activity (14 ms), whereas the Parsing Consent activity is the fastest (8 ms). The analysis of the GENERAL_D performance evidenced that the extra time, due to the GENERAL_D process, i.e., converting the consent into enforceable ACP, can be negligible considering the overall computation time of the MiMurcia’s infrastructure.

## 7. Conclusions and Discussions

This paper focused on evolving the currently adopted Smart City architecture to assure the *by-design* enforcement of different GDPR provisions. Therefore, we proposed a privacy-preserving system that ensures the management and tracking of personal data in a straightforward, user-centric, and user-friendly manner. The proposal relies on identity proving, authentication and privacy mechanisms (i.e., zero-knowledge proofs) for users’ identity management. An instantiation of the enhanced architecture has also been proposed, integrating an available Consent Manager (CM), a GDPR-based Access Control (AC), and a smart city IoT platform in compliance with the GDPR.

As evidenced in the state-of-the-art analysis (Section 2), the currently available proposals cannot fully satisfy the needs of a privacy-by-design, user-centric architecture of SCs. The experience of this paper allows us to collect the mains needs and requirements in enhancing existing proposals to achieve a privacy-friendly SC architecture. We can summarize them into:

- availability of personal data processing facilities able to manage specific data processing purposes;
- availability of facilities for collecting and managing Data Subjects consents (e.g., allowing giving and withdrawing those consents);
- availability of secure and privacy-preserving enforcement of the consent given by the Data Subject.

Thus, despite its preliminary conceptualization, the proposal of this paper has been able to address several issues about data privacy protection in the currently available implementations of the SCs. It evidences the necessity of effective and efficient enforcement of lawful processing of personal data that circulates inside the SCs. As evidenced in this paper, the adopted *by-design* solution could be one of the most effective ways for reducing privacy risks and increasing users’ trust in the global SC environment. Further, the preliminary performance results have shown the feasibly of the proposal and encouraged further investigation on the baseline idea.

One of the main threats to validity of the proposed architecture is the evaluation of the overall security level. Discovering the criticalities of a system is always a valid mean for putting in practice efficacious and corrective actions to improve its overall security. This is extremely true and important for Access Control Systems (ACSs) (both ACPs and ACMs) because their security and privacy vulnerabilities could insert the risk of releasing inadequate security solutions that allow unauthorized access (*security perspective*) or allowing unlawful processing of personal data (*legal perspective*). For this purpose, as future work, we are working to include in the architecture the Validation and Testing component of the GENERAL_D architecture.

The take-home message of our current work can be summarized into the following guidelines:

1. the SCs developers or providers should provide facilities for implementing the definition of specific purpose, collection of data, and specification of consents for processing personal data;
2. the SCs should include the enforcement of the access to personal data;
3. the SCs should provide easy-to-use and user-friendly facilities for letting the data subject exercising her/his rights;
4. the SCs should define, implement and integrate enhanced privacy-preserving systems that increase the usability and trust across the SC services;
5. the SCs should ensure stakeholders user-centric and user-friendly management and tracking of their personal data.

## Data Availability

Data sharing not applicable.

## Abbreviations

The following abbreviations are used in this manuscript:

ABC4Trust	Attribute-based Credentials for Trust
AC	Access Control
ACM	Access Control Mechanism
ACP	Access Control Policy
ACS	Access Control System
API	Application Programming Interface
ARIES	ReliAble euRopean Identity EcoSystem
B2C	Business-to-Consumer
CM	Consent Manager
CoAP	Constrained Application Protocol
DLT	Distributed Ledger Technology
DPV	Data Privacy Vocabulary
EU	European Union
GDPR	General Data Protection Regulation
GENERAL_D	Gdpr-based ENforcEment of peRsonAL Data
GIS	Geographic Information System
GPS	Global Positioning System
ICT	Information and Communication Technology
IdM	Identity Management
IdP	Identity Provider
IoT	Internet of Things
IP	Internet Protocol
JSON	JavaScript Object Notation
MAC	Media Access Control
MQTT	Message Queuing Telemetry Transport
NGSI	Next Generation Service Interfaces
OLYMPUS	ObLivious identitY Management for Private and User-friendly Services
PA	Pubilc Administration
p-ABC	Privacy-preserving Attribute-Based Credential
PAP	Policy Administration Point
PDP	Policy Decision Point
PEP	Policy Enforcement Point
SC	Smart City
SIS	Smart ICT System
SME	Small and Medium-sized Enterprise
TaCIoT	Trust-aware access control system for IoT
vIdP	Virtual Identity Provider
XACML	eXtensible Access Control Markup Language
